# Enhanced Capacitive Performance of Microwave-Driven CNTs on Carbonized Cigarette Filter Waste for Sustainable Energy Storage

**DOI:** 10.3390/nano15040257

**Published:** 2025-02-08

**Authors:** Young Joong Choi, Damin Lee, Se-Hun Kwon, Kwang Ho Kim

**Affiliations:** 1 School of Materials Science and Engineering, Pusan National University, Busan 46241, Republic of Korea; yjchoi0782@pusan.ac.kr; 2 Regional Leading Research Center for Smart Energy System, Kyungpook National University, Daegu 41566, Republic of Korea; damin91@knu.ac.kr; 3 Global Frontier R&D Center for Hybrid Interface Materials, Pusan National University, 2 Busandaehak-ro 63 beon-gil, Geumjeong-gu, Busan 46241, Republic of Korea

**Keywords:** cigarette filter waste, nitrogen-doped carbon nanotubes, microwave synthesis, sustainable electrode materials, supercapacitors

## Abstract

Microplastic pollution represents a significant global environmental issue, with cigarette filters being a major contributor due to their slow biodegradation. To address this issue while creating valuable materials, we developed a novel approach to synthesize nitrogen-doped carbon nanotubes on carbonized cigarette filter powder (NCNT@cCFP) using a microwave irradiation and nickel-catalyzed process. The successful incorporation of nitrogen (~6.6 at.%) and the enhanced graphitic structure create a hierarchical conductive network with abundant active sites for electrochemical reactions. The resulting NCNT@cCFP electrode exhibits a specific capacitance of 452 F/g at 1 A/g in a three-electrode configuration. The integrated hierarchical structure facilitates efficient electron transport and ion diffusion, leading to excellent rate capability (91.6% at 10 A/g) and cycling stability (96.5% retention after 5000 cycles). Furthermore, a symmetric supercapacitor device demonstrates promising energy storage capability with a maximum energy density of 14.0 Wh/kg at 483.1 W/kg, while maintaining 10.4 Wh/kg at a high power density of 4419.1 W/kg. This synergistic waste recycling strategy combined with microwave-driven synthesis offers a sustainable pathway for developing high-performance energy storage materials.

## 1. Introduction

Microplastic pollution poses a significant global environmental challenge, with cigarette filters (CFs) being a major contributor due to their slow biodegradation and persistence in the environment [1,2]. Composed primarily of cellulose acetate (CA), CFs represent one of the most commonly discarded waste items, with an estimated 5.5 trillion units disposed of globally each year [3]. The extremely slow biodegradation of these filters results in long-term environmental persistence, during which they fragment into microplastics and release toxic chemicals, posing serious risks to ecosystems and public health [4,5,6]. The accumulation of non-biodegradable CFs in marine environments is of particular concern, as they can persist for decades while continuously releasing harmful substances. However, the high carbon content and unique fibrous structure of CFs present an opportunity to repurpose these materials into functional products, particularly for energy storage applications, offering a potential solution to the environmental burden of CF waste [7,8,9]. Among the various potential applications, carbon-based supercapacitors stand out as a promising avenue for utilizing recycled CFs. These devices are characterized by their high power density, rapid charge–discharge rates, and long-term cycling stability, making them ideal candidates for energy storage technologies [10,11,12,13,14]. Although previous studies have demonstrated the feasibility of converting CFs into functional carbon materials through carbonization, these materials often exhibit poor electrochemical performance in supercapacitors, primarily due to limitations in conductivity and surface area [15,16,17,18]. Direct carbonization, while simple, does not fully exploit the structural potential of the CA precursor. Consequently, advanced modification strategies are necessary to enhance the functional properties of these materials for high-performance energy storage applications. One promising approach to improving the performance of carbon-based supercapacitor electrodes is the incorporation of carbon nanotubes (CNTs). CNTs are well regarded for their unique tubular structure, which facilitates efficient electron transport and provides a high density of active sites for charge storage [19,20,21,22,23]. Their one-dimensional architecture supports rapid charge transfer along the length of the tubes, while their high aspect ratio and large surface area enhance electrolyte interactions. In addition to these structural advantages, CNTs exhibit excellent mechanical strength and chemical stability, making them ideal candidates for high-performance energy storage devices. However, traditional CNT synthesis methods often require complex procedures, high energy consumption, and reliance on expensive catalysts or toxic chemicals, which limits their scalability and environmental sustainability [24,25,26]. Microwave-assisted synthesis has emerged as a promising technique for producing advanced carbon nanomaterials with superior properties and simplified processing. Previous studies have largely focused on using commercial conductive substrates; however, applying this method to recycled materials has not been extensively explored [27,28,29,30]. Microwave irradiation enables rapid CNT formation under ambient conditions, offering significant advantages in terms of energy efficiency and process simplicity. This technique also demonstrates versatility, suggesting the potential for sustainable material development when applied to recycled substrates. These features make microwave-assisted synthesis particularly attractive for the development of cost-effective and environmentally friendly nanomaterials, with considerable potential for scalable energy storage technologies.

In this study, we present a sustainable approach to repurposing CF waste into high-performance supercapacitor electrodes. We use a deacetylated and carbonized CF powder as a substrate for CNT growth via a nickel-catalyzed microwave irradiation process. Nitrogen-doped carbon nanotubes (NCNTs) were rapidly synthesized using azobis(cyclohexanecarbonitrile) as both the carbon source and nitrogen precursor. The microwave irradiation induces rapid heating, facilitating the growth of CNTs on the surface of the carbonized CF powder (cCFP), offering advantages over traditional chemical vapor deposition (CVD) methods in terms of simplicity, cost effectiveness, and environmental sustainability. Raman spectroscopy analysis revealed a reduced I_D_/I_G_ ratio for the NCNT-grown cCFP (NCNT@cCFP), confirming the formation of uniformly distributed NCNTs with enhanced graphitization and fewer structural defects. Additional chemical bonding analyses confirmed successful nitrogen incorporation (~6.6 at.%) and an increased sp^2^/sp^3^ ratio, resulting in a hierarchical conductive network with abundant active sites. The specific capacitance of the pristine cCFP was 257 F/g, whereas the NCNT@cCFP exhibited a significantly higher capacitance of 452 F/g. The enhanced electrochemical performance can be attributed to the hierarchical structure of NCNT@cCFP, which improves conductivity and charge storage capacity. Furthermore, cycle stability tests demonstrated excellent long-term performance, with minimal capacitance decay over 5000 cycles. When assembled into a symmetric supercapacitor, NCNT@cCFP electrodes demonstrate promising performance with a maximum energy density of 14.0 Wh/kg at 483.1 W/kg, while maintaining 10.4 Wh/kg at a high power density of 4419.1 W/kg. By comparing the electrochemical performance of cCFP and NCNT@cCFP, this study highlights the significant improvements in conductivity and capacitance achieved through NCNT integration, as validated by electrochemical and impedance analyses. This work not only addresses the environmental burden of CF waste but also leverages microwave-assisted synthesis to create a scalable and cost-effective solution for the development of high-performance electrode materials, contributing to the advancement of sustainable energy storage technologies.

## 2. Experimental Methods

### 2.1. Deacetylation and Carbonization Processes for cCFP

CFs were carefully separated from discarded cigarette butts by removing the outer paper wrapping and residual tobacco. The collected CFs were initially cleaned to remove any surface contaminants. To convert the CA-based CFs into cellulose via deacetylation, the cleaned CFs were immersed in a 0.25 M sodium hydroxide (NaOH) solution at room temperature for 4 h without stirring. The deacetylated CFs were then thoroughly washed with deionized water until neutral pH was achieved and dried in a vacuum oven at 50 °C for 10 h. The carbonization process was performed in a quartz tubular furnace under an Ar atmosphere. The dried, deacetylated CFs were heated to 450 °C at a controlled heating rate of 2 °C/min and maintained at this temperature for 1 h. The temperature was then increased to 900 °C at the same heating rate and held for 3 h to ensure complete carbonization. After natural cooling to room temperature under the Ar atmosphere, the carbonized material was collected and ground into a fine powder using a mortar and pestle to obtain the cCFP.

### 2.2. Microwave-Induced Growth of NCNT on cCFP

Nickel acetate tetrahydrate (Ni(CH_3_COO)_2_·4H_2_O) was employed as a catalyst precursor, and azobis(cyclohexanecarbonitrile) (ABCN) served as both the carbon and nitrogen sources for NCNT growth. Initially, the nickel acetate tetrahydrate and cCFP were mixed in a mass ratio of 1:10 in 3 mL of ethanol under sonication for 30 min to ensure uniform coating of the catalyst precursor. The mixture was then dried in a vacuum oven at 50 °C until the solvent was completely evaporated, yielding nickel acetate-coated cCFP. Subsequently, 60 mg of ABCN was thoroughly mixed with the catalyst-coated cCFP and placed in a quartz crucible. Microwave-assisted CNT growth was conducted using a conventional microwave oven (800 W) for 120 s under ambient conditions. After natural cooling for 20 min, the resultant black powder was collected and denoted as NCNT@cCFP.

### 2.3. Material Characterizations

The surface morphology and microstructure of the samples were examined using field emission scanning electron microscopy (FE-SEM, Hitachi S-4800, Hitachi, Ltd., Tokyo, Japan) operated at an acceleration voltage of 10 kV and an emission current of 7 μA. The microstructure and elemental mapping analysis were performed using transmission electron microscopy (TEM, JEM-2100F KBSI yeongnam regional center, JEOL Ltd., Tokyo, Japan) operating at an acceleration voltage of 200 kV. Raman spectroscopy (InVia, RENISHAW, Renishaw, UK) was employed to evaluate the degree of graphitization and structural properties of the carbon materials. The specific surface area and pore characteristics were investigated using nitrogen adsorption–desorption measurements (Nanoporosity-XQ) with a Brunauer–Emmett–Teller (BET) analysis. The chemical states and elemental composition of the samples were analyzed by X-ray photoelectron spectroscopy (XPS, Thermo Fisher Scientific K-ALPHA +XPS System, Thermo Fisher, San Diego, CA, USA) at 12 kV.

### 2.4. Electrochemical Measurements

Working electrodes were prepared by mixing the active materials (cCFP or NCNT@cCFP), carbon black, and polyvinylidene fluoride in a mass ratio of 8:1:1 in N-methyl-2-pyrrolidone to form a homogeneous slurry. The slurry was then uniformly coated onto carbon fiber and dried to obtain the working electrode. Electrochemical measurements were conducted using a 6 M KOH aqueous solution as the electrolyte in a three-electrode configuration. Platinum (Pt) and Hg/HgO electrodes served as the counter and reference electrodes. Electrochemical performance was evaluated using an electrochemical workstation (VMP3, BioLogic). Cyclic voltammetry (CV) measurements were conducted within a potential window of 0 to −1.0 V at various scan rates. Galvanostatic charge–discharge (GCD) tests were performed at different current densities (1, 2, 3, 5, and 10 A/g) in the same potential range. Electrochemical impedance spectroscopy (EIS) was conducted in the frequency range from 0.1 Hz to 100 kHz with an amplitude of 5 mV at open circuit potential. Long-term cycling stability was evaluated by continuous GCD measurements at 10 A/g. For symmetric supercapacitor tests, NCNT@cCFP electrodes were assembled in a coin cell configuration using a 6 M KOH electrolyte. The electrodes were prepared following the same coating method described above. The symmetric supercapacitor performance was evaluated in a potential window of 0–1.0 V at various scan rates (5–50 mV/s) and current densities (1–10 A/g).

## 3. Results and Discussion

**Figure 1:**
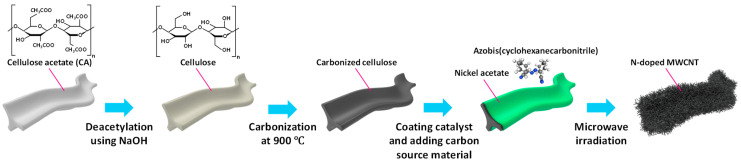
Schematic procedure for forming cigarette-filter-driven carbon filter powder (cCFP) and NCNT@cCFP using a microwave irradiation process.

Figure 1 illustrates the systematic process for converting waste CFs into high-performance electrode materials through a combination of chemical treatment and microwave irradiation synthesis. The process begins with the deacetylation of CA-based CFs using NaOH treatment, which converts the acetyl groups (−OCOCH_3_) to hydroxyl groups (−OH), resulting in a cellulose structure. This deacetylation step is crucial, as direct carbonization of untreated CFs leads to structural collapse, failing to maintain the fibrous morphology. The conversion to cellulose through deacetylation not only preserves the original fiber structure during carbonization but also enhances the thermal stability of the material during subsequent high-temperature treatment. Moreover, this specific concentration of NaOH treatment (0.25 M) facilitates the natural formation of porous structures during the carbonization process as the deacetylated cellulose undergoes thermal decomposition [31]. These inherent pores provide additional active sites and enhance the surface area of the carbonized material. The carbonization process at 900 °C transforms the deacetylated cellulose into a conductive carbon framework while maintaining its original fibrous morphology. This two-step heating process, first to 450 °C for 1 h, followed by heating to 900 °C for 3 h, ensures complete carbonization and the development of a well-defined carbon structure. The resulting cCFP serves as an ideal substrate for subsequent CNT growth. For the growth of NCNTs, we employed a novel approach utilizing nickel acetate as a catalyst precursor and ABCN as both carbon and nitrogen sources. This strategy offers several advantages over conventional ferrocene-based methods. The separation of the catalyst and carbon–nitrogen sources allows for better control over the CNT growth process, while ABCN enables in situ nitrogen doping during CNT formation. The microwave irradiation synthesis provides rapid and energy-efficient CNT growth under ambient conditions, eliminating the need for complex vacuum systems or specialized atmospheres typically required in traditional CVD methods. The final NCNT@cCFP product represents a hierarchical carbon structure where NCNTs are uniformly grown on the cCFP surface. This unique architecture combines the advantages of both the conductive cCFP substrate and the high surface area provided by the NCNT network, making it particularly suitable for energy storage applications.

**Figure 2:**
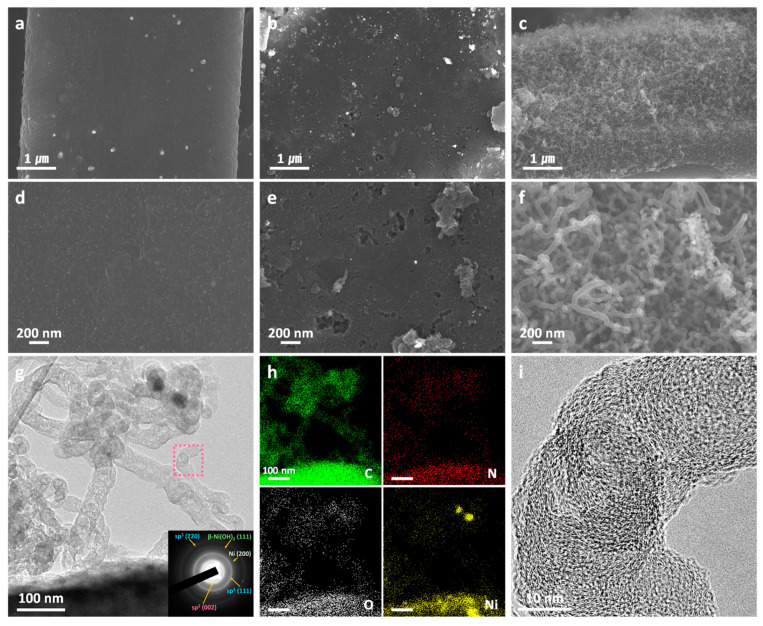
Morphological and structural characterization of the NCNT@cCFP through the Ni-catalyzed microwave-assisted process. (**a**,**d**) Pristine cCFP. (**b**,**e**) Ni-coated cCFP after microwave treatment without carbon source. (**c**,**f**) Hierarchical NCNT structures are grown on cCFP through a microwave irradiation process with ABCN. (**g**) TEM image of the NCNT@cCFP with corresponding selected area electron diffraction (SAED) pattern (inset). (**h**) Elemental mapping images showing the distribution of C, N, O, and Ni in the NCNT@cCFP. (**i**) High-resolution TEM image of the marked area (pink dotted square) in (**g**).

The FE-SEM analysis investigated the surface characteristics at different stages of CNT growth: the pristine cCFP substrate, the intermediate stage without the nitrogen-containing carbon source, and the final NCNT@cCFP with the catalyst and ABCN. Figure 2a,d present the surface morphology of the pristine cCFP at different magnifications. Due to the deacetylation of the CFs, the cCFP maintains its original fibrous structure after the high-temperature carbonization process, demonstrating the effectiveness of our deacetylation treatment in preserving structural integrity. The surface of these carbonized fibers appears smooth and uniform without significant surface features, confirming the successful transformation into a carbonaceous material without structural collapse. A distinct morphological change was observed after the microwave treatment without ABCN (Figure 2b,e). The surface exhibits extensive oxidation features and irregular structures resulting from intense discharge phenomena during microwave irradiation. In the absence of the nitrogen-containing carbon precursor, continuous intense arc discharges occur, leading to severe oxidation of the nickel acetate on the cCFP surface. This intermediate stage clearly demonstrates the critical role of ABCN in controlling the CNT growth process by moderating the discharge intensity and subsequent reaction pathways. After introducing ABCN with the nickel catalyst, the morphology underwent a dramatic transformation (Figure 2c,f), showing uniform coverage of intertwined NCNT networks. The high-magnification images show that the NCNTs exhibited diameters ranging from 35 to 60 nm, with the majority falling within the 35–50 nm range, forming a well-interconnected network structure. To further investigate the internal structure of these NCNTs, a TEM analysis was conducted. The TEM image (Figure 2g) clearly shows the successful growth of CNT structures on the cCFP surface. The corresponding selected area electron diffraction (SAED) pattern (inset) reveals the predominant presence of sp^2^-hybridized carbon along with minor contributions from sp^3^ carbon and β-Ni(OH)_2_, confirming the formation of graphitic CNT structures with residual catalyst. The elemental mapping analysis (Figure 2h) demonstrates the uniform distribution of C, N, O, and Ni throughout the NCNT structure, providing direct evidence of successful nitrogen doping and the presence of remaining Ni catalyst in the form of β-Ni(OH)_2_. The high-resolution TEM image (Figure 2i), taken from the marked area (pink dotted square) in Figure 2g, reveals well-defined graphitic structures of the CNTs with clear lattice fringes, further confirming their highly crystalline nature. This hierarchical architecture, combining the cCFP substrate with a dense NCNT network, provides both efficient electron transport pathways and an increased surface area for charge storage. The uniform distribution of NCNTs across the cCFP surface was attributed to the optimized ratio between the nickel catalyst and ABCN, which prevented the formation of carbon aggregates commonly observed when using single-source precursors such as ferrocene.

**Figure 3:**
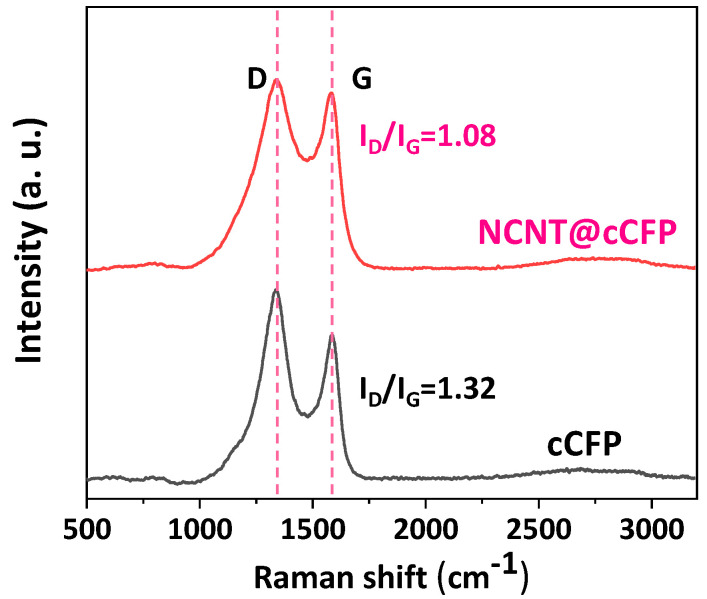
Raman spectra comparing cCFP and NCNT@cCFP.

Raman spectroscopy was conducted to confirm CNT formation on the cCFP surface and to evaluate the structural quality of the resulting materials. As shown in Figure 3, both the cCFP and NCNT@cCFP exhibit prominent peaks at approximately 1342 cm^−1^ (D band) and 1588 cm^−1^ (G band), which are characteristic of carbon-based materials. The G band, associated with the in-plane vibrations of sp^2^ carbon atoms, reflects the presence of more ordered graphitic domains, while the D band indicates structural defects and disorder. The intensity ratio (I_D_/I_G_) provides additional insight into the degree of graphitization. For cCFP, the I_D_/I_G_ ratio is 1.32, indicative of the substantial disorder commonly observed in carbonized-biomass-derived materials. In contrast, after the microwave-assisted growth of nitrogen-doped CNTs, the NCNT@cCFP sample exhibits a significantly lower I_D_/I_G_ ratio of 1.08, suggesting the formation of a more graphitic and structurally ordered CNT network. Notably, this enhanced graphitic character is achieved despite incorporating nitrogen heteroatoms, which typically introduce additional defects. These findings confirm the successful synthesis of high-quality NCNTs and underscore the effectiveness of our microwave-assisted growth strategy. To investigate the physical properties of the hierarchical porous structure, nitrogen adsorption–desorption measurements were performed (Figure S1). The cCFP shows a typical type I isotherm with a sharp uptake at low relative pressures (P/P_0_ < 0.1), suggesting a well-developed microporous structure with a specific surface area of 467.87 m^2^/g. After growing the NCNTs, the NCNT@cCFP exhibits combined type I and IV isotherm characteristics, where the additional adsorption at high relative pressures (P/P_0_ > 0.8) indicates the formation of mesopores. The BJH pore size distribution reveals that both samples maintain predominantly microporous features, while the NCNT@cCFP shows additional mesopores attributed to the interconnected NCNT network structure. The specific surface area of the NCNT@cCFP is 359.47 m^2^/g, which is slightly lower than that of the cCFP due to the modification of some micropores during NCNT growth. However, the hierarchical architecture combining micropores with highly conductive NCNT networks provides more efficient electron–ion transport pathways, which can contribute to enhanced electrochemical performance despite the decreased specific surface area. To further evaluate the electrochemically active surface area (EASA), CV measurements were conducted in the non-Faradaic region (−0.2 to 0.2 V) at various scan rates (Figure S2). The double-layer capacitance (C_dl_) was determined from the slope of the linear relationship between the average current and scan rate, and the EASA values were calculated as shown in Table S1. The results demonstrate that the NCNT@cCFP electrode exhibits an enhanced electrochemically accessible surface area (163.2 m^2^/g) compared to the cCFP (69.6 m^2^/g), contributing to its superior capacitive performance.

**Figure 4:**
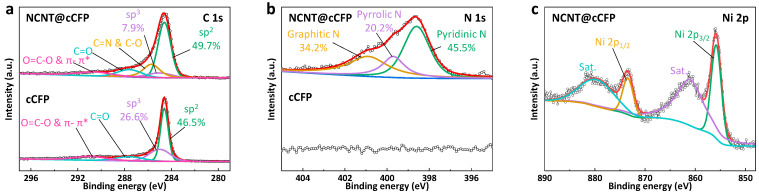
XPS analysis comparing the chemical states and bonding configurations between the NCNT@cCFP and cCFP. (**a**) C 1s spectra of the NCNT@cCFP and cCFP. (**b**) N 1s spectra of the NCNT@cCFP and cCFP. (**c**) High-resolution Ni 2p spectrum of the NCNT@cCFP.

The chemical states and bonding configurations of the NCNT@cCFP and cCFP were systematically investigated using XPS analysis. The C 1s spectra (Figure 4a) reveal a pronounced structural evolution in the NCNT@cCFP, as evidenced by a significant increase in the sp^2^/sp^3^ ratio from 1.8 in the cCFP to 6.3 in the NCNT@cCFP. Notably, the dominance of the sp^2^ carbon peak at 284.6 eV [32] and the diminished sp^3^ carbon contribution at 285.0 eV [33] clearly indicate a shift toward a more graphitic structure. In terms of absolute percentages, the NCNT@cCFP shows an increased sp^2^ carbon content (49.7% vs. 46.5% in cCFP) and a substantial decrease in sp^3^ carbon (7.9% vs. 26.6%), underscoring the enhanced graphitization achieved through NCNT growth and improved electrical conductivity. Additional peaks corresponding to C=N and C–O (285.7 eV) [32], C=O and N–C=O (287.6 eV) [34], C=O and N–C=O (287.6 eV) [34], and O–C=O and π–π* (290.6 eV) [35] suggest the presence of various functional groups that may facilitate better electrolyte interactions and ion transport. The successful nitrogen incorporation (~6.6 at.%) is evidenced by the N 1s spectrum (Figure 4b), which displays three distinct nitrogen configurations in the NCNT@cCFP: pyridinic N (45.5% at 398.6 eV) [36], pyrrolic N (20.2% at 399.7 eV) [36], and graphitic N (34.2% at 400.9 eV) [36,37]. The predominance of pyridinic and graphitic nitrogen indicates effective nitrogen doping, altering the electronic structure of the carbon framework to enhance conductivity and introduce additional electrochemically active sites. The Ni 2p spectrum (Figure 4c) confirms that the residual nickel catalyst remains as Ni(OH)_2_, as indicated by the Ni 2p_3/2_ peak at 855.8 eV [38,39] with a satellite at 861.0 eV and the Ni 2p_1/2_ peak at 873.4 eV [38,39] with a satellite at 879.6 eV. Given that Ni constitutes only ~2 at.% in the NCNT@cCFP sample, the corresponding Ni(OH)_2_ amount adds a negligible inactive mass, resulting in minimal impact on the electrochemical performance of the electrode. Moreover, retaining this small amount of Ni(OH)_2_ obviates the need for acid washing, thereby preventing hazardous waste generation and potential structural damage to the NCNT network. This approach supports our sustainable, simplified synthesis strategy without compromising electrode integrity or performance. These XPS analyses confirm the successful formation of NCNTs with improved conductivity, abundant active sites, and a stable catalyst residue managed without additional processing steps. Such structural and chemical enhancements provide a solid foundation for understanding the superior electrochemical performance of the NCNT@cCFP electrodes.

**Figure 5:**
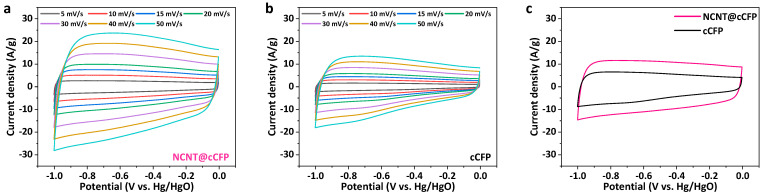
Electrochemical analysis of NCNT@cCFP and cCFP electrodes. CV curves at various scan rates for (**a**) NCNT@cCFP and (**b**) cCFP, respectively. (**c**) Comparison of cyclic voltammetry curves between NCNT@cCFP and cCFP at 25 mV/s.

The electrochemical properties of the cCFP and NCNT@cCFP electrodes were first evaluated using CV measurements. Figure 5a shows the CV curves of NCNT@cCFP at various scan rates from 5 to 50 mV/s. The NCNT@cCFP electrode exhibits quasi-rectangular CV curves with a more significant current density, maintaining its shape even at high scan rates, indicating predominantly EDLC behavior and excellent rate capability. For comparison, Figure 5b presents the CV curves of cCFP at the same scan rates, showing a significantly lower current response. The direct comparison of both electrodes at 25 mV/s (Figure 5c) clearly demonstrates the superior electrochemical performance of the NCNT@cCFP over cCFP. Notably, the CV curve of the cCFP shows redox peaks at −0.6~−0.8 V, which can be attributed to surface oxygen functional groups remaining from incomplete carbonization. In contrast, the NCNT@cCFP electrode displays a more EDLC-like behavior with a quasi-rectangular shape. This behavior primarily results from the significantly enhanced electrical conductivity provided by the NCNT network, which facilitates rapid electron transport and enables more efficient double-layer charge storage. While faradaic reactions from heteroatoms still contribute to the total capacitance, the substantial enhancement in electrical conductivity leads to a dominant EDLC characteristic in the CV curves. The improved electrochemical performance of the NCNT@cCFP can be attributed to the synergistic combination of structural advantages. The well-integrated NCNT network facilitates efficient electron conduction throughout the electrode while increasing the accessibility of active sites for ion storage. Additionally, the improved electrical conductivity, resulting from the higher degree of graphitization confirmed by Raman analysis and XPS, further contributes to the superior capacitive performance and rate capability of the NCNT@cCFP electrode.

**Figure 6:**
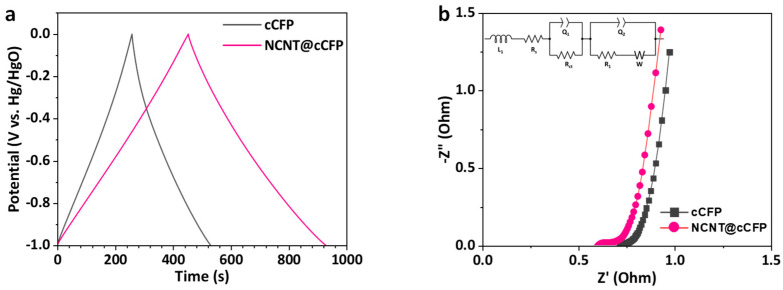
Evaluation of charge storage behavior and resistances. (**a**) GCD curves of cCFP and NCNT@cCFP at 1 A/g. (**b**) Nyquist plots of cCFP and NCNT@cCFP electrodes.

To understand the charge storage behavior and electrochemical kinetics of the electrodes, GCD measurements and EIS were conducted. Figure 6a shows the GCD curves of the cCFP and NCNT@cCFP electrodes at a current density of 1 A/g. The NCNT@cCFP electrode exhibits a significantly longer discharge time compared to the cCFP, indicating higher charge storage capacity. Based on these GCD curves, the specific capacitance was calculated to be 452 F/g for the NCNT@cCFP, which is substantially higher than the 257 F/g obtained for the cCFP. This remarkable enhancement in capacitance can be attributed to the synergistic effects of the hierarchical NCNT network, which provides more accessible pathways for ion diffusion, improves the conductive connections between the carbon domains, and introduces additional active sites through nitrogen doping via the ABCN precursor. The charge transfer kinetics and electrical conductivity of the electrodes were further investigated using EIS measurements. The Nyquist plots of both electrodes (Figure 6b) consist of a semicircle in the high-frequency region and a nearly vertical line in the low-frequency region, characteristic of capacitive behavior. In this analysis, R_s_ corresponds to the ohmic resistance, which includes the intrinsic resistance of the electrode, electrolyte, and electrode–electrolyte interface, while R_ct_ represents the charge transfer resistance, reflecting the ease of electron exchange at the electrode–electrolyte interface [40]. The mid-frequency region involves the pore resistance (R_1_) associated with ion transport through the porous network of the electrode material and its corresponding non-ideal capacitive behavior(Q_2_) [41]. The NCNT@cCFP electrode shows a lower R_s_ (0.597 Ω) and R_ct_ (0.1274 Ω) compared to the cCFP (R_s_ = 0.693 Ω, R_ct_ = 1.723 Ω), indicating enhanced electrical conductivity and more efficient charge transfer kinetics, respectively. This improvement can be attributed to both the effective formation of the CNT-based conductive network and the nitrogen-doping strategy, which provides additional active sites and improves the wettability of the carbon surface.

**Figure 7:**
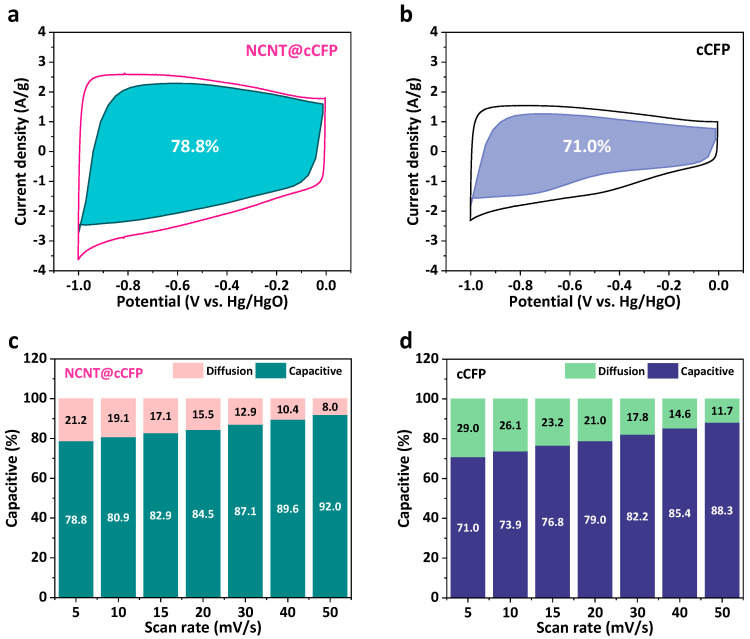
Analysis of capacitive and diffusion-controlled contributions. CV curves showing capacitive and diffusion regions for (**a**) NCNT@cCFP and (**b**) cCFP at 5 mV/s. Relative contributions at different scan rates for (**c**) NCNT@cCFP and (**d**) cCFP.

We analyzed the capacitive and diffusion-controlled contributions to the total capacitance to gain deeper insights into the charge storage mechanism. Figure 7a,b presents the CV curves with separated capacitive contributions (shaded regions) for both the NCNT@cCFP and cCFP electrodes at 5 mV/s. The NCNT@cCFP electrode exhibits a significantly higher capacitive contribution of 78.8% compared to 71.0% for the cCFP. This increased capacitive contribution is attributed to the enhanced electrical conductivity from the NCNT network, which promotes rapid surface-controlled charge storage processes. It is worth noting that diffusion-controlled processes (including faradaic reactions from heteroatoms) still contribute to the total capacitance, but their relative proportion appears smaller due to the substantially enhanced capacitive contribution. The evolution of capacitive contributions with increasing scan rates was further analyzed (Figure 7c,d). Both electrodes show an increasing trend in capacitive contribution as the scan rate increases from 5 to 50 mV/s, suggesting that surface-controlled processes become more dominant at higher scan rates. Notably, NCNT@cCFP maintains consistently higher capacitive portions across all scan rates, ranging from 78.8% at 5 mV/s to 92.0% at 50 mV/s. In contrast, cCFP shows relatively lower capacitive contributions, increasing from 71.0% at 5 mV/s to 88.3% at 50 mV/s. This superior capacitive behavior of NCNT@cCFP can be attributed to its hierarchical NCNT-integrated structure, which facilitates more efficient ion and electron transport to active sites, enabling rapid charge storage processes even at higher scan rates. 

**Figure 8:**
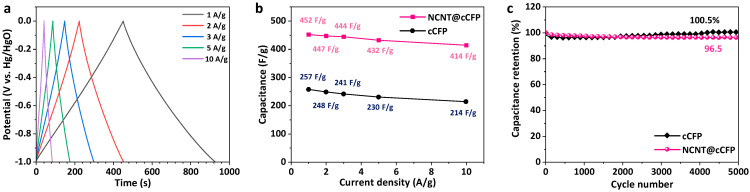
Rate capability and cycling stability of the electrodes. (**a**) GCD curves of the NCNT@cCFP at different current densities (1–10 A/g). (**b**) Rate capability comparison between the NCNT@cCFP and cCFP. (**c**) Cycling stability of the NCNT@cCFP and cCFP at 10 A/g for 5000 cycles.

The rate performance and long-term cycling stability of the electrodes were evaluated to assess their practical applicability. Figure 8a shows the GCD curves of the NCNT@cCFP at different current densities from 1 to 10 A/g, maintaining approximately symmetrical triangular shapes, indicative of near-ideal capacitive behavior. As shown in Figure 8b, the NCNT@cCFP demonstrates superior rate capability, retaining 91.6% of its initial capacitance (414 F/g at 10 A/g compared to 452 F/g at 1 A/g) when the current density increases by a factor of ten. In contrast, the cCFP exhibits a steeper capacitance decrease, maintaining only 83.1% of its initial value (214 F/g at 10 A/g from 257 F/g at 1 A/g). The enhanced rate performance of the NCNT@cCFP can be attributed to its hierarchical structure, where NCNTs provide efficient pathways for both electron transport and ion diffusion. To contextualize the performance of the NCNT@cCFP, we compared its specific capacitance with previously reported carbon-based electrodes tested under similar conditions (Table S2). The specific capacitance of 452 F/g achieved by the NCNT@cCFP at 1 A/g demonstrates competitive performance among reported carbon-based electrodes, highlighting the effectiveness of our microwave-assisted synthesis approach. The long-term cycling stability was investigated by continuous GCD cycling at 10 A/g for 5000 cycles (Figure 8c). Notably, the cCFP demonstrates excellent cycling stability, slightly increasing to about 100.5% of its initial capacitance after 5000 cycles, while the NCNT@cCFP maintains 96.5% retention. Although the NCNT@cCFP undergoes a minor decrease in capacitance over prolonged cycling, its overall retention remains notably high (96.5%), underscoring the effectiveness of the nitrogen-doped CNT network in maintaining both structural integrity and electrochemical activity. Furthermore, when tested at a higher current density of 20 A/g for an extended period of 10,000 cycles, the NCNT@cCFP maintained 98.5% of its initial capacitance (Figure S3), demonstrating exceptional long-term stability. This stable cycling performance may be attributed to improved electrode–electrolyte interface characteristics provided by the hierarchical NCNT architecture, which ensures efficient ion transport and preserves active sites, sustaining superior electrochemical performance even after numerous charge–discharge cycles.

**Figure 9:**
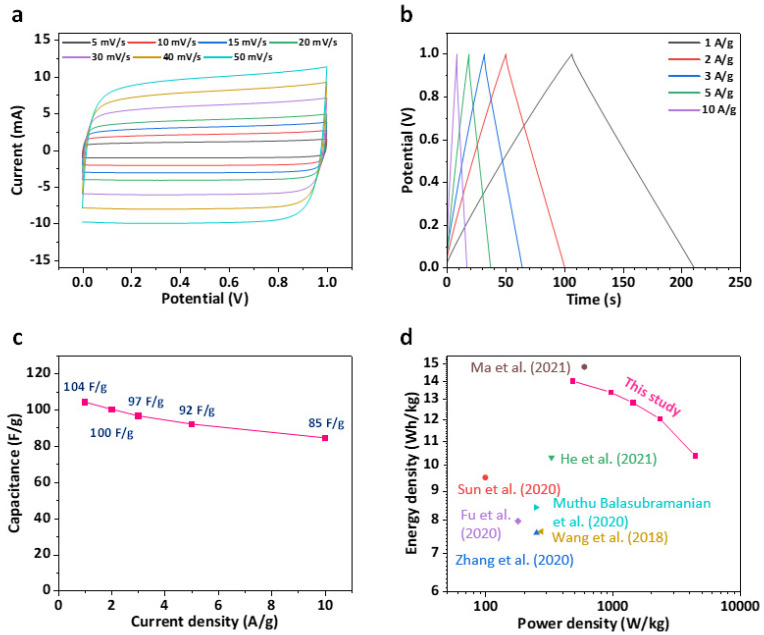
Electrochemical analysis of symmetric supercapacitor device assembled with NCNT@cCFP. (**a**) CV curves at different scan rates from 5 to 50 mV/s. (**b**) GCD profiles at various current densities. (**c**) Rate capability with increasing current density. (**d**) Ragone plot comparing energy and power densities with previously reported carbon-based supercapacitors [42,43,44,45,46,47,48].

To evaluate the practical applicability of the NCNT@cCFP for energy storage applications, we assembled and tested a symmetric supercapacitor. Figure 9a shows the CV curves of the device at different scan rates from 5 to 50 mV/s within a potential window of 0 to 1.0 V. The quasi-rectangular shape of the CV curves with no prominent redox peaks indicates typical EDLC behavior, while their preserved shape, even at high scan rates, suggests excellent rate capability. The GCD curves at various current densities (Figure 9b) exhibit nearly triangular and symmetric shapes, further confirming the ideal capacitive characteristics of the device. Based on the discharge curves, the specific capacitance was calculated to be 104 F/g at 1 A/g. As shown in Figure 9c, the device demonstrates good rate performance, maintaining 85 F/g (81.7% retention) even at a high current density of 10 A/g. The energy and power density characteristics of the device were evaluated using a Ragone plot (Figure 9d). The symmetric supercapacitor delivers a maximum energy density of 14.0 Wh/kg at a power density of 483.1 W/kg, while maintaining 10.4 Wh/kg at a high power density of 4419.1 W/kg. These values are competitive with or superior to previously reported carbon-based supercapacitors, demonstrating the potential of our NCNT@cCFP electrodes for practical energy storage applications [42,43,44,45,46,47,48]. The superior device performance can be attributed to the synergistic effects of the hierarchical NCNT network structure, which provides efficient pathways for both electron transport and ion diffusion, while nitrogen doping enhances the electrical conductivity and wettability of the electrode surface.

## 4. Conclusions

In summary, we have demonstrated an effective strategy for converting CF waste into high-performance supercapacitor electrodes through microwave-driven NCNT growth. Our modified carbonization process preserves the fibrous structure of CFs, producing a stable carbon scaffold that provides a suitable substrate for subsequent NCNT growth and enhances electrochemical activity. The subsequent microwave irradiation synthesis, utilizing nickel acetate as a catalyst and ABCN as a dual carbon–nitrogen source, enables rapid NCNT formation under ambient conditions, offering significant advantages in energy efficiency and process simplicity. The XPS analysis confirms a higher sp^2^/sp^3^ ratio and successful nitrogen incorporation (~6.6 at.%), indicating improved graphitization and additional active sites. The NCNT@cCFP electrode exhibits remarkable electrochemical performance, with a high specific capacitance of 452 F/g at 1 A/g, significantly outperforming the pristine cCFP (257 F/g). This superior performance can be attributed to the hierarchical NCNT-integrated structure, which not only improves electron and ion transport but also increases the number of electrochemically active sites, thereby enhancing charge storage efficiency. The improved graphitization and nitrogen doping further enhance conductivity and electrode–electrolyte interactions. Furthermore, the NCNT@cCFP demonstrates excellent rate capability, with 91.6% capacitance retention at 10 A/g, and maintains 96.5% of its initial capacitance after 5000 cycles, highlighting its long-term stability. The extended cycling test at 20 A/g demonstrates exceptional stability with 98.5% capacitance retention after 10,000 cycles. When assembled into a symmetric supercapacitor configuration, the device delivers a maximum energy density of 14.0 Wh/kg at 483.1 W/kg while maintaining 10.4 Wh/kg at a high power density of 4419.1 W/kg. This approach provides a sustainable solution for both CF waste recycling and high-performance energy storage material development. The strategy presented here could potentially be extended to other waste-derived carbon materials, offering new possibilities for sustainable energy storage applications.

## Data Availability

The original contributions presented in this study are included in the article/Supplementary Materials. Further inquiries can be directed to the corresponding author(s).

## Supplementary Materials

The following supporting information can be downloaded at: https://www.mdpi.com/article/10.3390/nano15040257/s1, Figure S1: N_2_ adsorption-desorption isotherms with pore size distribution (inset) of (a) cCFP and (b) NCNT@cCFP for surface area analysis; Figure S2: CV measurements for double-layer capacitance (C_dl_) calculation. (a) CV curves of cCFP at different scan rates in the non-Faradaic region. (b) CV curves of NCNT@cCFP at different scan rates in the non-Faradaic region. (c) Linear fitting of the average current versus scan rate to determine the C_dl_ of cCFP and NCNT@cCFP; Figure S3: Extended cycling performance of NCNT@cCFP at 20 A/g for 10,000 cycles; Table S1: Calculation results of electrochemically active surface area (EASA) for NCNT@cCFP and cCFP electrodes. The EASA values were determined from the measured double-layer capacitance (C_dl_) and specific capacitance (C_s_); Table S2: Comparison of electrochemical performance between NCNT@cCFP and previously reported carbon-based supercapacitor electrodes.
