# Supplementary material for: Enhanced Capacitive Performance of Microwave-Driven CNTs on Carbonized Cigarette Filter Waste for Sustainable Energy Storage
Source: Nanomaterials (Basel). 2025 Feb 8;15(4):257. doi: 10.3390/nano15040257 (PMC11858238; doi:10.3390/nano15040257)
Supplement: Supplementary file 1 [file nanomaterials-15-00257-s001.zip › nanomaterials-3413371-supplementary.pdf]

Supporting Information

Enhanced Capacitive Performance of Microwave-Driven CNTs on Carbonized Cigarette Filter Waste for Sustainable Energy Storage

Young Joong Choi [1,†], Damin Lee [2,†], Se-Hun Kwon [1,*] and Kwang Ho Kim [3,*]

[1] School of Materials Science and Engineering, Pusan National University, Busan 46241, Republic of Korea; yjchoi0782@pusan.ac.kr
[2] Regional Leading Research Center for Smart Energy System, Kyungpook National University, Daegu 41566, Republic of Korea; damin91@knu.ac.kr
[3] Global Frontier R&D Center for Hybrid Interface Materials, Pusan National University, 2 Busandaehak-ro 63 beon-gil, Geumjeong-gu, Busan 46241, Republic of Korea
* Correspondence: sehun@pusan.ac.kr (S.-H.K.), kwhokim@pusan.ac.kr (K.H.K.)
† These authors contributed equally to this work.

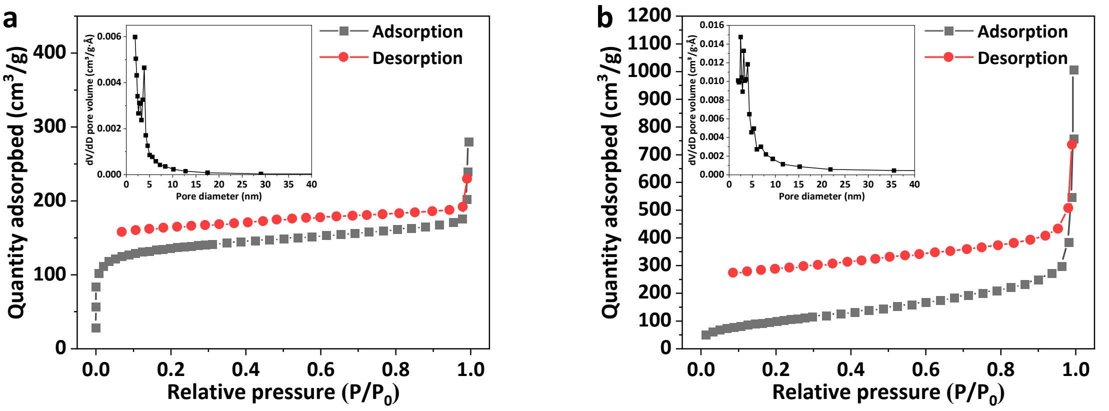

Figure S1. N_2 adsorption-desorption isotherms with pore size distribution (inset) of (**a**) cCFP and (**b**) NCNT@cCFP for surface area analysis.

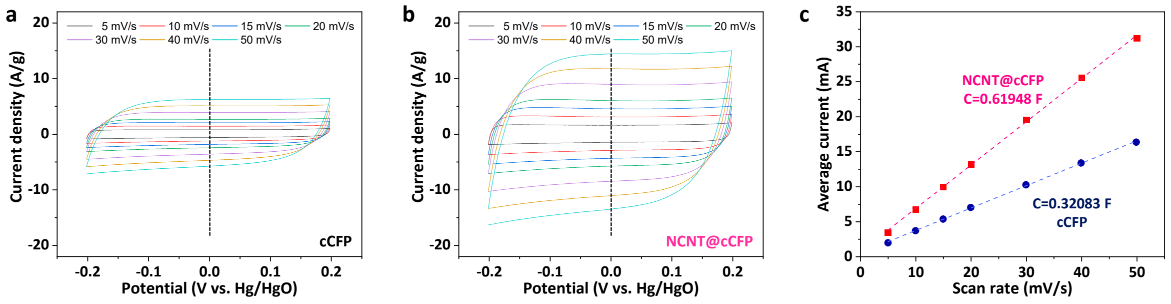

Figure S2. CV measurements for double-layer capacitance (C_{dl}) calculation. (**a**) CV curves of cCFP at different scan rates in the non-Faradaic region. (**b**) CV curves of NCNT@cCFP at different scan rates in the non-Faradaic region. (**c**) Linear fitting of the average current versus scan rate to determine the C_{dl} of cCFP and NCNT@cCFP.

Table S1. Calculation results of electrochemically active surface area (EASA) for NCNT@cCFP and cCFP electrodes. The EASA values were determined from the measured double-layer capacitance (C_{dl}) and specific capacitance (C_s).

Electrodes	C_{dl} (F)	C_s (μF /cm^2)	EASA(m^2/g)
NCNT@cCFP	0.61948	170 [1]	163.2
cCFP	0.32083	170 [1]	69.6

The C_s was determined using reference values from previous research on carbonized cigarette filters without an activation process [18]. Using this reference value (C_s = 170 μF/cm^2) along with our measured C_{dl}, the EASA (m^2/g) was calculated according to the following equation:

$$EASA = C_{dl}/(C_s \times m)$$

Where C_{dl} is the measured double-layer capacitance (F) from Figure S1c, C_s is the specific capacitance (μF/cm^2), and m is the mass of the active material (g).

Table S2. Comparison of electrochemical performance between NCNT@cCFP and previously reported carbon-based supercapacitor electrodes.

Electrodes	Potential window (V)	C (F /g)	Current density	Electrolyte	Ref.
QPC-3	1.0	330	1 A/g	6M KOH	[42]
SF-PC700-3	1.0	530	0.5 A/g	6M KOH	[43]
NCS-650	1.0	324	1 A/g	6M KOH	[44]
WS-600	1.2	262.7	0.5 A/g	6M KOH	[45]
APC	1.0	220.6	0.5 A/g	6M KOH	[46]
PC-CNTs	0.8	208.8	1 A/g	6M KOH	[47]
$ZBTC_{0.8}$-900	1.2	352	1 A/g	6M KOH	[48]
**NCNT@cCFP**	**1.0**	**451.7**	**1 A/g**	**6M KOH**	**This study**

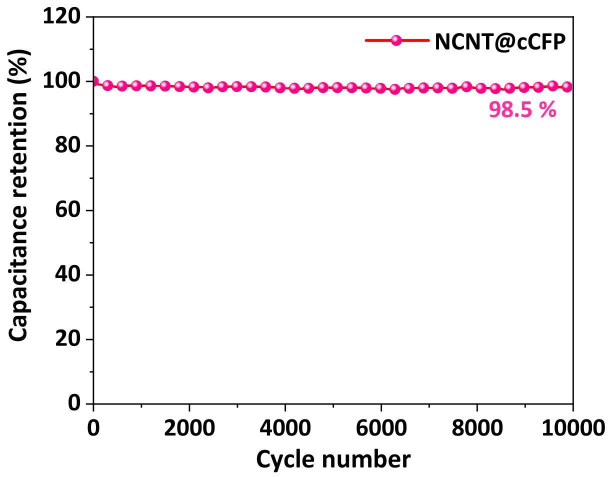

Figure S3. Extended cycling performance of NCNT@cCFP at 20 A/g for 10,000 cycles.
